# Socio-economic inequalities in the chronic diseases management among Chinese adults aged 45 years and above: a cross sectional study

**DOI:** 10.1186/s13690-021-00678-1

**Published:** 2021-08-30

**Authors:** Jing Guo, Jiasen Li, Kehui Huang, Ning Huang, Xing Lin Feng

**Affiliations:** Department of Health Policy and Management, School of Public Health, Peking University, Beijing, People’s Republic of China

**Keywords:** Socio-economic, Inequalities, Social capital, Chronic disease

## Abstract

**Backgrounds:**

Non-communicable diseases (NCDs) have become a priority public health issue. The aim of this study was to examine whether socio-economic inequalities exist in chronic disease management among Chinese adults, and whether the relationship between SES and chronic disease management mediated by social capital.

**Methods:**

We used combined data from China Health and Retirement Longitudinal Study (CHARLS). A total of 19,291 subjects, including 14,905 subjects from 2011 survey, 2036 subjects from 2013, and 2350 subjects from 2015 was included in this study.

**Results:**

Subjects living in urban setting, with higher education attainment and economic status were more likely to have annual health checks, and to be diagnosed for those with hypertension, diabetes and dyslipidemia (all *P* < 0.05). Social participation could mediate the association between social economic status (SES) and annual health checks, diagnosis of hypertension and dyslipidemia, and health education of hypertension. Health checks could mediate the association between social participation and the diagnosis of hypertension, diabetes and dyslipidemia. The proportions of mediation were 17.5, 23.9 and 8.9%, respectively. There were no mediating effects observed from cognitive social capital variable-perceived helpfulness.

**Conclusion:**

It is necessary to deeply reform our social security system and enhance the social capital construction to promote those low SES people’s physical health.

## Background

Non-communicable diseases (NCDs) have become a priority public health issue [1, 2]. With an annual toll of 41 million, NCDs accounted for 71% of the global mortality burden. Among these causes, 17.5 million (46.2%) were attributed to cardiovascular diseases and 1.5 million (4%) were attributed to diabetes mellitus [3]. The pandemic of NCDs disproportionally affect low- and middle- income countries (LMICs), which suffered from fragmented and deficit health systems [2, 4].

Similarly, China bears enormous burden of NCDs, contributing to the largest number of morbidity and mortality [5, 6]. Data from national surveillance reported that the morbidity of single and multiple NCD, in Chinses population, was 64.7% and 53.4%, respectively, and major NCDs attributed to 86.6% of total deaths in China [7, 8]. With China’s rapid economic development, behavioral factors, including tobacco and alcohol consumption, exercise, and obesity, and modifiable factors such as raising blood pressure and blood glucose were reported as major risk factors leading to the pandemic of NCDs at an unprecedented pace [9], with the deteriorating effects further aggregated by the rapid ageing process. Thus, it is necessary to better manage non-communicable diseases, and reduce the burden of chronic disease in China.

World Health organizations (WHO) has published a global plan for the prevention and control of NCD, and recommended community-based intervention for chronic conditions management such as hypertension, diabetes and hyperlipidemia [10]. However, fragmented care is commonplace, and the insufficient resources are challenged within the current health system [11]. A previous study reported that appropriately 44% to 76% cases of the three chronic conditions went undiagnosed among Chinese adults aged 45 and older during 2011 to 2013, in spite of the National Public Health Service Program that freely provides some screening and routine management of NCDs since 2009 [4]. This indicated that the management of NCDs in China has largely become a case identification problem. Using a national representative sample of China, through mediation analyses, we attempted to explore the potential influence of social capital on the relationship between SES and chronic diseases management.

### Conceptual framework

**Fig. 1:**
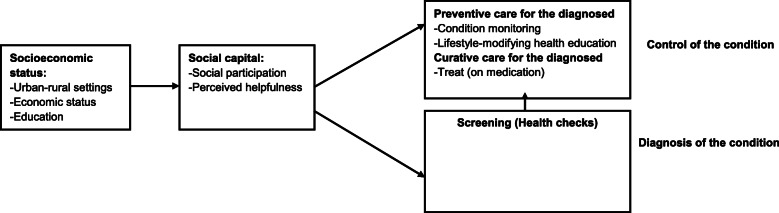
Conceptual framework for the chronic diseases management

Figure 1 showed the conceptual framework of the potential pathways that social capital might mediate the relationship between SES and the health management of hypertension, diabetes, and dyslipidemia. On one hand, SES are very important influencing factors for health and management of NCDs, which also lead to the inequity of health [12–15]. On the other hand, social capital mediates the relationship between SES inequality and health status [16]. Individuals with low SES hold less social resources and poor health status, and SES may influence their health through pathways of social networking and social support from family members and friends [17]. In addition, according to the social cohesion theory, social capital, as a contextual concept, requires multilevel analysis of its influence on health [18]. A common distinction of social capital is between cognitive and structural dimensions. Cognitive social capital includes values and attitudes such as norms of reciprocity and trust in others, while the structural component reflects observable aspects of social organization such as social networks or civic engagement [19]. Social participation as structural social capital examines the extent of associations and activity in society, which could influence health status through behavioral processes such as control of risk behavior and better adherence in medical treatment [20]. However, previous studies mostly focus on the structural dimension of social capital, and draw few attentions to the cognitive dimension of social capital. Perceived helpfulness as cognitive social capital shows to what extent individuals can rely on the support of others in the case of illness [21]. This study will include two dimensions of social capital, structural and cognitive dimensions.

The aim of this study was to examine whether socio-economic inequalities exist in chronic disease management among Chinese adults, and whether the relationship between SES and chronic disease management is mediated by social capital.

## Material and methods

### Study design

We used combined data from China Health and Retirement Longitudinal Study (CHARLS) 2011 national baseline, 2013 follow-up surveys and 2015 follow-up surveys. The CHARLS baseline survey in 2011 is a nationally representative survey of Chinese adults aged 45 and older, which also oversampled candidates for further follow-ups [22]. The CHARLS adopted a four-stage stratified Probability Proportional to size (PPS) random sampling procedure to yield nationally representative samples of people older than 45 years [23]. At the first stage (county level), all counties/districts were firstly stratified by province, urbanity and economic development, and then 150 counties/districts were randomly selected proportionally. At the second stage, three villages/communities were randomly chosen from each county as primary sampling units (PSUs). At the third stage, 80 households were randomly selected as candidates, in each of the 450 PSUs, where 24 were investigated in the baseline and others were reserved as refreshing candidates for further follow-ups. At the fourth stage, one random person older than 45 years was selected. And both this individual and his/her spouse were interviewed using structured questionnaires. Of note, the CHARLS is a longitudinal study, and thus many samples were the same in the three waves of surveys. However, the CHARLS added households from the baseline reservations as refreshed samples in the recent two surveys. The 2011 baseline survey investigated a total of 10,257 households and 17,414 individuals. The 2013 follow-up survey added 2726 individuals as refreshed samples. 3707 individuals in 2015 follow-up survey not investigated in 2011 and 2013 surveys were used as refreshed samples. And the criterion of refreshed samples selection is whether they are 45–46-year-olds, in order to avoid underrepresentation of the sample aged 45+ due to the sample respondents getting older in the next wave. After excluding missing data, finally, a total of 19,291 subjects, including 14,905 subjects from 2011 survey, 2036 subjects from 2013, and 2350 subjects from 2015 was included in this study.

The CHARLS has several modules. Despite the anthropometric measurements which was carried out by trained nurses, data on participants’ socio-demographic background and their utilization of health care were collected in face-to-face interviews by trained students using the Computer-Assisted Interviewing System with details described elsewhere [23] Peking University administered the CHARLS and data are released to the public for research use upon application [23]. The founders play no role in interpreting the data.

In our study, socio-demographic and anthropometric data were released for the 2011 national survey, the 2013 and 2015 follow-up surveys, while blood biochemical data was released only for the 2011 survey. And the participants with, saying hypertension, should have already been acknowledged his/her condition during the baseline survey. Therefore, we extracted the full sample from the 2011 survey and the refreshed sample from the 2013 and 2015 survey to analyze the health checks and hypertension outcomes and used only the 2011 survey to analyze outcomes for diabetes and dyslipidemia. And including the 2013 and 2015 data could not only enlarge the sample size, but also provide new data. Besides, we have done sensitivity analyses by excluding the 2013 and 2015 refreshed samples, the mediating effects of social capital do not differ. Thus, we could believe such a treat will not affect the interpretation of the data.

### Measures

#### Dependent variables

Chronic disease management.

##### Health check

Each respondent was asked whether he/she received any health checks in the previous year.

##### Hypertension management

For the anthropometric measurements, a trained nurse used an Omron TM HEM- 7112 electronic monitor to measure each interviewee’s systolic blood pressure (SBP) and diastolic blood pressure (DBP) three times. The mean SBP and DBP values were then calculated. A subject was defined as having hypertension if he/she had a mean SBP ≥ 140 mmHg and/or DBP ≥ 90 mmHg or reported use of antihypertensive medication [10]. In the interviews, the respondents were also asked, “Have you been diagnosed with hypertension by a doctor?” For those who answered “Yes,” further questions were asked, including whether they received any lifestyle-modifying interventions from a health professional and whether they monitored their blood pressure in the previous year. Treated hypertension was defined as the use of anti-hypertensive medication for elevated blood pressure. An individual with controlled hypertension was defined as a hypertensive person with a mean SBP < 140 mmHg and DBP < 90 mmHg.

##### Diabetes management

Regarding the blood biochemical assessment, medically trained staff collected three tubes of venous blood from subjects to measure a set of clinical indicators based on standard protocol. Over 92% of respondents whose blood was drawn reported that they had fasted overnight. The following biochemical measures were analyzed: plasma glucose and HbA1c for diabetes and total cholesterol, total triglycerides, high-density lipoprotein cholesterol (HDL-C), and low-density lipoprotein cholesterol (LDL-C) for dyslipidemia. A subject was defined as having diabetes if he/she had a fasting plasma glucose level ≥ 126 mg/dL, or if he/she did not fast overnight, had an HbA1c ≥6.5%, or reported a doctor’s diagnosis [24]. For those who reported a doctor’s diagnosis of diabetes, further questions were asked, including whether they received any lifestyle-modifying interventions for this condition from a health professional and whether they monitored their conditions in the previous year. We used a diagnostic cutoff of HbA1c ≥ 6.5%, while defining diabetes control as HbA1c < 7.0% according to the Chinese national guideline [25].

##### Dyslipidemia management

A subject was defined as having dyslipidemia if he/she had a total cholesterol level ≥ 240 mg/dL, total triglycerides ≥200 mg/dL, HDL-C < 40 mg/dL, or LDL-C ≥ 160 mg/dL or reported a doctor’s diagnosis [26]. For those who reported a doctor’s diagnosis of dyslipidemia, further questions were asked, including whether they received any lifestyle-modifying interventions for this condition from a health professional and whether they monitored their conditions in the previous year.

#### Independent variables: socioeconomic status (SES)

##### Education

Years of education were used to measure education attainment.

Urban or rural was also used as an indicator of SES, according to previous study [13].

##### Income

We used living expenditures to evaluate economic status because they tend to be less affected by information bias and can better capture respondents’ permanent income, especially for the retired elderly without any temporal income [19, 27]. The survey asked a couple of questions on household living expenditures. Households recalled food expenditures for the last week, and expenditures on communication, utilities, fuel, housekeeping, transportation, daily items and entertainment for the last month. Households were also asked expenditures for clothing, long distance travel, heating, furniture and durable goods, education, fitness, beauty, vehicle maintenance, taxes, automobiles, electronics, property management and donations in the last year. We aggregated these cost data to calculate each household’s annual living expenditures and then divided the number of heads in each household to calculate the per capita household living expenditures. The Consumer Price Index was used to adjust all monetary values for inflation relative to 2015 to enable comparison between years [28]. For living expenditures, the ln(x + 1) form was used in the following analysis.

#### Mediator variables: social capital

Social participation was the most commonly proxy variable to access social capital in the previous studies [21, 29, 30]. Social participation was defined as the numbers of these 8 activities took part in. More details can be seen in Table 2.

Perceived helpfulness was measured by this question: “If you needed help with basic daily activities like eating or dressing. Do you have relatives or friends (besides your spouse/partner) who would be willing and able to help you over a long period of time?” If the respondents answer “yes”, the variable was code as 1 and 0 otherwise. This definition was similar with previous studies [31, 32].

##### Covariates

The following demographic variables were included in the analyses as confounders: age (45–60; 61–75; > 75), gender (female; male), marital status (have a spouse/ do not have a spouse), social health insurance (New rural cooperative medical scheme and urban basic medical insurance/urban employee basic medical insurance and government insurance scheme/none), smoke (never/quit/still smoke) and drink (Never/ ≤ 1 time per month/ > 1 time per month).

##### Operational definition

Social capital was defined as the structural and cognitive social capital individuals had. Among them, structural social capital was measured by social participation, namely the number of social activities in the last month; cognitive social capital was measured by Perceived helpfulness, namely the experience of having relatives or friends (besides your spouse/partner) help individuals when they need help with basic daily activities like eating or dressing.

Socioeconomic status (SES) included three indicators, namely years of education, urban-rural hukou, and living expenditures. Individuals with urban hukou or more years of education or higher living expenditures, had higher socioeconomic status; people with rural hukou or less year of education or lower living expenditures, had lower socioeconomic status.

Education and Economic status in Table 1 were treated as ordinal variables with three categories, to describe the distribution of sample’s education and economic status. Education status was divided into Illiterate, Primary and Secondary and above. Economic status referred to three categories that living expenditures before taking logarithms was divided into, <$2, $2–$4 and ≥ $4.

**Table 1:** Characteristics of Chinese adults aged 45 years and above by urban-rural setting (*N* = 19,291)

Variable	Rural		Urban		Total	
	n	%	n	%	n	%
**Year**						
2011	8969	80.4	5936	72.9	14,905	77.3
2013	1080	9.7	956	11.7	2036	10.5
2015	1100	9.9	1250	15.4	2350	12.2
**Age**						
45–60	6640	59.6	4874	59.9	11,514	59.7
61–75	3687	33.1	2637	32.4	6324	32.8
> 75	822	7.4	631	7.7	1453	7.5
**Gender**						
Male	5484	49.2	3860	47.4	9344	48.4
Female	5665	50.8	4282	52.6	9947	51.6
**Marital status**						
Living alone	1957	17.6	1254	15.4	3211	16.6
Living with spouse	9192	82.4	6888	84.6	16,080	83.4
**Education**						
Illiterate	5282	47.4	2154	26.5	7436	38.6
Primary	2950	26.5	1856	22.8	4806	24.9
Secondary and above	2917	26.2	4132	50.7	7049	36.5
**Economic status**^a^						
< $2	5085	45.6	1931	23.7	7016	36.4
$2–$4	3436	30.8	2258	27.7	5694	29.5
≥ $4	2628	23.6	3953	48.6	6581	34.1
**Social health insurance**^b^						
NCMS&URBMI	10,195	91.4	4804	59.0	14,999	77.8
UEBMI&GIS	300	2.7	2485	30.5	2785	14.4
No coverage	654	5.9	853	10.5	1507	7.8
**Smoke**						
Never	6600	59.2	5099	62.6	11,699	60.6
Quit	935	8.4	774	9.5	1709	8.9
Still smoke	3614	32.4	2269	27.9	5883	30.5
**Drink**						
Never	7314	65.6	5358	65.8	12,672	65.7
≤ 1 time per month	842	7.6	718	8.8	1560	8.1
> 1 time per month	2993	26.8	2066	25.4	5059	26.2

^a^ Daily household per capita living expenditures were used to measure economic status, adjusted for inflations relative to the year 2015. We used the exchange rate 1USD = 6.23RMB in the year 2015

^b^ NCMS, New Rural Cooperative Medical Scheme; URBMI, Urban Resident Basic Medical Insurance. UEBMI, Urban Employee Basic Medical insurance; GIS, government insurance scheme. These insurance schemes are grouped together because they provide similar benefit packages

#### Statistical analysis

Descriptive characteristics were calculated for the characteristics of sample. Because all of our dependent variables were dichotomous, all analyses employed the logistic model, and each analysis adjusted for the same potential confounding of age, gender, marital status, social health insurance, smoking and drinking. First, each indicator of socioeconomic status was separately entered into the logistic model with different measures of chronic disease management as dependent variables, in order to explore the relationship between social capital and chronic disease management. Second, each type of social capital was separately introduced into mediation analyses using logistic regression to examine the direct associations between SES and chronic disease management, as well as the indirect associations via social capital. In addition, to adequately evaluate the indirect and direct associations, we used the bootstrapping method, and obtained point estimates of the coefficients and their bootstrap 95% CI from bootstrap resamples. All analyses were conducted using Software for Statistics and Data Science (STATA) 14.0.

## Results

Among 19,291 samples, 77.3% (14, 905 out of 19, 291) were from 2011 national baseline survey, 10.5% (2036 out of 19, 291) from 2013 follow-up survey and 12.2% (2, 350 out of 19, 291) from 2015 follow-up survey. About 63.5% (12,242 out of 19, 291) subjects were either illiterate or had only primary education, and such proportion was much higher for subjects living in rural areas than subjects living in urban areas. In term of daily household per capita expenditures, 36.4% (7016 out of 19,291) subjects were less than $2, 29.5% (5694 out of 19,291) between $2 and $4, 34.1% (6581 out of 19,291) more than $4. 77.8% (14,999 out of 19,291) subjects were covered by NCMS and URBMI, 14.4% (2785 out of 19,291) by UEBMI and GIS, 7.8% (1507 out of 19,291) did not have any social health insurance (see Table 1).

Social participation was measured by 8 social activities taking last month. 49.1% (5477 out of 11,149) rural subjects and 55.5% (4520 out of 8142) urban subjects took at least one social activity last month, respectively. About 69.1% (13,333 out of 19,291) subjects thought they would have relatives and friends who would be willing and able to help them over a long period when they needed help with basic daily activities in the future. Such proportion were 70.3% (7834 out of 11,149) for rural residents and 67.5% (5499 out of 8142) for urban residents, respectively (see Table 2).

**Table 2:** The measurement of social participation and perceived helpfulness among Chinese adults aged 45 years and above by urban-rural setting ^a^

Variable	Rural		Urban		Total	
	n	%	n	%	n^b^	%^b^
**Social participation**						
Respondent who reported taking the following activities last month						
Any of the 8 following activities	5477	49.1	4520	55.5	9997	51.8
(1) Interacted with friends	4196	37.6	2923	35.9	7119	36.9
(2) Played Ma-jiang, chess and cards or went to community club	1951	17.5	1902	23.6	3853	20.0
(3) Provided help to family, friends, or neighbors who did not live with you and who did not pay you for help	1087	9.7	837	10.3	1924	10.0
(4) A sport, social, or other kind of club	220	2.0	1246	15.3	1466	7.6
(5) A community-related organization	112	1.0	280	3.4	392	2.0
(6) Done voluntary or charity work	58	0.5	133	1.6	191	1.0
(7) Cared for a sick or disabled adult who did not live with you and who did not pay you for help	133	1.2	150	1.8	283	1.5
(8) An educational or training course	31	0.3	90	1.1	121	0.6
**Perceived helpfulness**						
Yes	7834	70.3	5499	67.5	13,333	69.1
No	3314	29.7	2643	32.5	5958	30.9

^a^ There were 6.7 and 4.5% subjects missing in measuring the constructs of social participation and perceived helpfulness, respectively. After dropping subjects with missing data, 19,291 subjects were included in this analysis. Among them, total number of rural and urban subjects was 11,149 and 8142, respectively

^b^ The number (n) in the column of “Total” means the total number of respondents with the “yes” response to corresponding question, while the percentage (%) in the column of “Total” means the proportion of them to the total sample size

Table 3 shows status quo in managing hypertension, diabetes, and dyslipidemia along the care continuum, among Chinese adults 45 years and above by urban-rural setting. 37.3% (3024 out of 8116) urban subjects had annual health checks, while only 28.9% (3213 out of 11,116) for rural subjects. As a whole, only 32.4% (6237 out of 19,232) subjects had annual health checks. Of the Chinese adults aged 45 and older with hypertension, 62.3% (4161 out of 6683) were diagnosed. Among subjects with diagnosed hypertension, 86.7% were monitored yearly, 59.3% got health education, 87.7% were on antihypertensive medications, and 35.1% had controlled hypertension. Of the Chinese adults aged 45 and older with diabetes in 2011, 50% (871 out of 1742) were diagnosed. Among subjects with diagnosed diabetes, 77.8% were monitored yearly, 76.0% got health education, 70.8% were on diabetes medications, and 69.0% had controlled diabetes. Of the Chinese adults aged 45 and older with dyslipidemia, 29.2% (1400 out of 4790) were diagnosed. Among subjects with diagnosed dyslipidemia, 54.0% were on dyslipidemia medications, 43.0% had controlled dyslipidemia. The performance gap in diagnosis of hypertension, diabetes and dyslipidemia, namely the difference between diagnosis and actual prevalence of these three chronic diseases, seemed to be the main problems in chronic disease management (see Table 3).

**Table 3:** Status in managing hypertension, diabetes, and dyslipidemia, among Chinese adults 45 years and above by urban-rural setting

Dependent variables	Rural		Urban		Total	
	n^a^	%	n^a^	%	n^a^	%
**Health checks**						
**Yes**	3213	28.9	3024	37.3	6237	32.4
**No**	7903	71.1	5092	62.7	12,995	67.6
**Hypertension**						
Diagnosis^b^	2122	58.0	2039	67.5	4161	62.3
None diagnosis	1540	42.0	982	33.5	2522	26.7
*Among diagnosed*						
Condition monitoring^c^	1839	86.7	1767	86.7	3606	86.7
Health education^d^	1156	54.5	1311	64.3	2467	59.3
On medication	1823	85.9	1828	89.7	3651	87.7
Control^e^	630	29.7	575	28.2	1205	30.0
**Diabetes**						
Diagnosis^b^	380	40.8	491	60.6	871	50.0
None diagnosis	552	59.2	319	39.4	871	50.0
*Among diagnosed*						
Condition monitoring^c^	276	72.6	402	81.9	678	77.8
Health education^d^	273	71.8	389	79.2	662	76.0
On medication	258	67.9	359	73.1	617	70.8
Control^e^	210	55.2	220	44.8	430	49.4
**Dyslipidemia**						
Diagnosis^b^	629	23.6	771	36.4	1400	29.2
None diagnosis	2041	72.4	1349	73.6	3390	70.8
*Among diagnosed*						
On medication	348	55.3	408	52.9	756	54.0
Control^e^	249	39.6	182	23.6	431	30.8

^a^ n is the numerator

^b^ For hypertension, “Diagnosis” refers to a subject who had a mean SBP ≥140 mmHg and/or DBP ≥90 mmHg or reported use of antihypertensive medication. For diabetes, “Diagnosis” refers to a subject who had a fasting plasma glucose level ≥ 126 mg/dL, or an HbA1c ≥6.5% if he/she did not fast overnight, or reported a doctor’s diagnosis of diabetes. For dyslipidemia, “Diagnosis” refers to a subject who had a total cholesterol level ≥ 240 mg/dL, or total triglycerides ≥200 mg/dL, or HDL-C < 40 mg/dL, or LDL-C ≥ 160 mg/dL or reported a doctor’s diagnosis of dyslipidemia

^c^ “Condition monitoring” referred to a subject who had a chronic condition and monitored his/her conditions in the past year. Condition monitoring refers to the monitoring of blood pressures for hypertension; and to the monitoring of blood sugar or HbA1c for diabetes

^d^ “Health education” refers to the uptake of lifestyle-modifying interventions from a health professional. In China, community health providers should provide “condition monitoring” and “health education” to the population-in their catchment area- who had a hypertension or diabetes free of charge

^e^ “Control” were defined as systolic blood pressure < 140 mmHg and diastolic blood pressure < 90 mmHg for hypertension, HbA1c < 7% for diabetes, total cholesterol < 240 mg/dL and total triglycerides < 200 mg/dL, and HDL-C ≥ 40 mg/dL and LDL-C < 160 mg/dL for dyslipidemia, respectively

As showed in Table 4, subjects living in urban setting, with higher education attainment and economic status were more likely to have annual health checks, and to be diagnosed for those with hypertension, diabetes, and dyslipidemia. Subjects with higher SES were more likely to take health education, and take antihypertensive medications amongst those with diagnosed hypertension. Subjects living in urban setting with higher economic status were more likely to be monitored yearly amongst those with diagnosed diabetes, but less likely to have controlled dyslipidemia. Subjects with higher education attainment were more likely to be monitored yearly for those with diagnosed hypertension. Subjects with higher economic status were more likely to have controlled hypertension, and to take diabetes medication for those with diagnosed hypertension and diabetes, respectively (see Table 4).

**Table 4:** Associations between socioeconomic status and the management of hypertension, diabetes, and dyslipidemia, among Chinese adults aged 45 years and above^a^

Dependent variables	Urban Setting (rural as reference)		Education		Economic status	
	OR	95% CI	OR	95% CI	OR	95% CI
**Health checks**	**1.246**	**(1.164, 1.333)**	**1.047**	**(1.037, 1.057)**	**1.087**	**(1.055, 1.121)**
**Hypertension**						
Diagnosis	**1.244**	**(1.114, 1.389)**	**1.033**	**(1.017, 1.050)**	**1.214**	**(1.155, 1.276)**
*Among diagnosed*						
Condition monitoring	0.960	(0.786, 1.173)	**1.039**	**(1.010, 1.068)**	1.083	(0.995, 1.178)
Health education	**1.382**	**(1.199, 1.593)**	**1.045**	**(1.024, 1.065)**	**1.194**	**(1.121, 1.272)**
On medication	**1.269**	**(1.029, 1.565)**	**1.043**	**(1.012, 1.074)**	**1.112**	**(1.017, 1.216)**
Control	1.002	(0.855, 1.173)	1.014	(0.992, 1.037)	**1.094**	**(1.018, 1.177)**
**Diabetes**						
Diagnosis	**1.672**	**(1.347, 2.075)**	**1.035**	**(1.004, 1.067)**	**1.277**	**(1.148, 1.420)**
*Among diagnosed*						
Condition monitoring	**1.718**	**(1.191, 2.478)**	1.044	(0.993, 1.097)	**1.222**	**(1.024, 1.458)**
Health education	1.252	(0.880, 1.781)	1.001	(0.954, 1.051)	1.126	(0.946, 1.341)
On medication	1.231	(0.882, 1.720)	1.023	(0.978, 1.070)	**1.194**	**(1.013, 1.407)**
Control	0.964	(0.660, 1.409)	1.002	(0.950, 1.057)	1.130	(0.938, 1.361)
**Dyslipidemia**						
Diagnosis	**1.344**	**(1.166, 1.549)**	**1.075**	**(1.053, 1.096)**	**1.214**	**(1.131, 1.303)**
*Among diagnosed*						
On medication	1.230	(0.960, 1.036)	0.978	(0.947, 1.009)	1.001	(0.893, 1.123)
Control	**0.556**	**(0.416, 0.742)**	0.989	(0.953, 1.027)	**0.867**	**(0.754, 0.998)**

^a^ Logistic regression were conducted, adjusting for age, sex, marital status, smoke, drink, social health insurance. Economic status was measured by annual per capita living expenditure. Education was measured by years’ of education achievement. Bolded coefficients are statistically significant at the P level of 0.05

Table 5 illustrates the mediating effects of social capital, measured as social participation and perceived helpfulness, respectively, on the association between urban-rural setting and the management of the three types of chronic conditions amongst Chinese adults 45 years and above. Under the same analytical structure, Tables 6 and 7 illustrate the mediating effects of social capital on the effects of household income and participants’ educational achievement, respectively. The results consistently show that social participation takes a statistically significant role that mediates the effects of participants’ socio-economic status, whenever measured as urban-rural differences (Table 5), households’ income (Table 6), or participants’ educational achievement (Table 7), on the uptake of health checks and the diagnosis of chronic conditions. In particular, the indirect effects of social participation seem to be more profound in mediating the association between participants’ educational achievement and the diagnosis of the three conditions and particularly the condition monitoring of diabetes (see Table 7).

**Table 5:** Social capital as a mediating factor on the association between urban-rural setting and chronic diseases management, among Chinese adults aged 45 years and above^a^

Dependent variables	Indirect effect via social participation		Indirect effect via perceived helpfulness		Direct effect		Total effect of urban-rural setting	
	Coefficient	95% CI	Coefficient	95% CI	Coefficient	95% CI	Coefficient	95% CI
**Health checks**	**0.0028**	**(0.0018, 0.0046)**	0.0001	(−0.0002, 0.0006)	**0.0567**	**(0.0404, 0.0731)**	**0.0596**	**(0.0433, 0.0764)**
**Hypertension**								
Diagnosis	**0.0016**	**(0.0003, 0.0036)**	0.0006	(− 0.0007, 0.0024)	**0.0576**	**(0.0252, 0.0893)**	**0.0598**	**(0.0283, 0.0950)**
*Among diagnosed*								
Condition monitoring	0.0019	(−0.0003, 0.0054)	− 0.0012	(− 0.0040, 0.0006)	−0.0114	(− 0.0614, 0.0421)	−0.0107	(− 0.0608, 0.0398)
Health education	**0.0016**	**(0.0004, 0.0042)**	−0.0005	(−0.0025, 0.0002)	**0.0878**	**(0.0442, 0.1285)**	**0.0889**	**(0.0494, 0.1309)**
On medication	−0.0003	(−0.0029, 0.0017)	0.0004	(−0.0007, 0.0024)	**0.0653**	**(0.0094, 0.1166)**	**0.0654**	**(0.0083, 0.1170)**
Control	0.0005	(−0.0008, 0.0033)	0.0002	(−0.0004, 0.0018)	− 0.0002	(− 0.0459, 0.0473)	0.0005	(− 0.0449, 0.0475)
**Diabetes**								
Diagnosis	0.0018	(−0.0009, 0.0099)	−0.0001	(− 0.0031, 0.0022)	**0.1382**	**(0.0720, 0.1960)**	**0.1399**	**(0.0767, 0.2031)**
*Among diagnosed*								
Condition monitoring	0.0038	(−0.0076, 0.0229)	−0.0005	(− 0.0085, 0.0019)	**0.1450**	**(0.0420, 0.2269)**	**0.1483**	**(0.0564, 0.2354)**
Health education	0.0013	(−0.0019, 0.0125)	−0.0009	(− 0.0117, 0.0039)	0.0597	(− 0.0291, 0.1806)	0.0601	(− 0.0285, 0.1825)
On medication	−0.0004	(− 0.0085, 0.0015)	0.0002	(− 0.0021, 0.0063)	0.0573	(− 0.0270, 0.1397)	0.0571	(− 0.0262, 0.1419)
Control	0.0004	(−0.0027, 0.0081)	0.0007	(−0.0022, 0.0111)	−0.0111	(− 0.1127, 0.0909)	− 0.0100	(− 0.1134, 0.0895)
**Dyslipidemia**								
Diagnosis	**0.0018**	**(0.0003, 0.0051)**	0.0007	(− 0.0002, 0.0019)	**0.0780**	**(0.0365, 0.1081)**	**0.0805**	**(0.0393, 0.1097)**
*Among diagnosed*								
On medication	− 0.0009	(− 0.0045, 0.0009)	0.0003	(− 0.0013, 0.0048)	0.0573	(− 0.0329, 0.1076)	0.0567	(− 0.0307, 0.1069)
Control	−0.0003	(− 0.0051, 0.0018)	− 0.0001	(− 0.0042, 0.0034)	−0.1595	(− 0.2417, − 0.0857)	−0.1599	(− 0.2407, − 0.0835)

^a^ Adjusting for age, sex, marital status, smoke, drink, social health insurance. Bolded coefficients are statistically significant at the P level of 0.05

**Table 6:** Social capital as a mediating factor on the association between economic status and chronic diseases management, among Chinese adults aged 45 years and above^a^

Dependent variables	Indirect effect via social participation		Indirect effect via perceived helpfulness		Direct effect		Total effect of economic status	
	Coefficient	95% CI	Coefficient	95% CI	Coefficient	95% CI	Coefficient	95% CI
**Health checks**	**0.0062**	**(0.0039, 0.0082)**	0.0004	(−0.0000, 0.0010)	**0.0439**	**(0.0247, 0.0620)**	**0.0505**	**(0.0308, 0.0690)**
**Hypertension**								
Diagnosis	**0.0026**	**(0.0003, 0.0058)**	−0.0002	(− 0.0024, 0.0012)	**0.1145**	**(0.0840, 0.1455)**	**0.1169**	**(0.0865, 0.1480)**
*Among diagnosed*								
Condition monitoring	0.0046	(−0.0006, 0.0126)	0.0014	(−0.0005, 0.0063)	0.0428	(−0.0156, 0.0968)	0.0488	(−0.0053, 0.1033)
Health education	**0.0036**	**(0.0006, 0.0090)**	0.0005	(−0.0001, 0.0025)	**0.1032**	**(0.0648, 0.1453)**	**0.1073**	**(0.0676, 0.1485)**
On medication	−0.0013	(−0.0061, 0.0039)	− 0.0005	(− 0.0030, 0.0005)	**0.0658**	**(0.0259, 0.1388)**	**0.0640**	**(0.0226, 0.1350)**
Control	**0.0036**	**(0.0010, 0.0074)**	−0.0001	(−0.0010, 0.0005)	**0.0490**	**(0.0066, 0.0861)**	**0.0525**	**(0.0087, 0.0892)**
**Diabetes**								
Diagnosis	**0.0044**	**(0.0005, 0.0113)**	−0.0001	(− 0.0032, 0.0014)	**0.1341**	**(0.0695, 0.1914)**	**0.1384**	**(0.0731, 0.1950)**
*Among diagnosed*								
Condition monitoring	**0.0127**	**(0.0020, 0.0281)**	−0.0008	(− 0.0085, 0.0013)	0.0936	(− 0.0027, 0.1776)	**0.1055**	**(0.0109, 0.1916)**
Health education	0.0042	(−0.0011, 0.0175)	−0.0014	(− 0.0103, 0.0024)	0.0602	(− 0.0343, 0.1553)	0.0630	(− 0.0346, 0.1552)
On medication	−0.0019	(− 0.0131, 0.0034)	0.0003	(− 0.0016, 0.0081)	**0.0955**	**(0.0147, 0.2261)**	**0.0939**	**(0.0178, 0.2290)**
Control	0.0013	(−0.0098, 0.0134)	0.0002	(−0.0026, 0.0067)	0.0641	(−0.0376, 0.1693)	0.0657	(−0.0260, 0.1821)
**Dyslipidemia**								
Diagnosis	**0.0040**	**(0.0009, 0.0077)**	−0.0004	(−0.0024, 0.0005)	**0.1030**	**(0.0715, 0.1424)**	**0.1066**	**(0.0753, 0.1501)**
*Among diagnosed*								
On medication	−0.0020	(−0.0076, 0.0004)	0.0001	(−0.0014, 0.0044)	0.0027	(−0.0642, 0.0608)	0.0008	(−0.0666, 0.0596)
Control	−0.0007	(−0.0074, 0.0022)	− 0.0001	(− 0.0031, 0.0016)	−0.0766	(− 0.1715, − 0.0081)	−0.0773	(− 0.1794, − 0.0169)

^a^ Adjusting for age, sex, marital status, smoke, drink, social health insurance. Bolded coefficients are statistically significant at the P level of 0.05

**Table 7:** Social capital as a mediating factor on the association between educational achievement and chronic diseases management, among Chinese adults aged 45 years and above^a^

Dependent variables	Indirect effect via social participation		Indirect effect via perceived helpfulness		Direct effect		Total effect of education	
	Coefficient	95% CI	Coefficient	95% CI	Coefficient	95% CI	Coefficient	95% CI
**Health checks**	**0.0077**	**(0.0055, 0.0109)**	0.0000	(−0.0004, 0.0007)	**0.0933**	**(0.0718, 0.1128)**	**0.1011**	**(0.0805, 0.1204)**
**Hypertension**								
Diagnosis	**0.0044**	**(0.0007, 0.0091)**	0.0013	(−0.0000, 0.0040)	**0.0671**	**(0.0248, 0.1040)**	**0.0728**	**(0.0292, 0.1085)**
*Among diagnosed*								
Condition monitoring	**0.0064**	**(0.0001, 0.0162)**	0.0000	(−0.0015, 0.0152)	**0.0796**	**(0.0107, 0.1385)**	**0.0860**	**(0.0200, 0.1428)**
Health education	**0.0054**	**(0.0007, 0.0126)**	0.0000	(−0.0014, 0.0011)	**0.0940**	**(0.0512, 0.1375)**	**0.0995**	**(0.0579, 0.1412)**
On medication	−0.0022	(−0.0108, 0.0052)	− 0.0000	(− 0.0019, 0.0014)	**0.0980**	**(0.0374, 0.1472)**	**0.0958**	**(0.0433, 0.1480)**
Control	**0.0057**	**(0.0017, 0.0115)**	0.0001	(−0.0003, 0.0012)	0.0259	(−0.0273, 0.0780)	0.0317	(−0.0172, 0.0852)
**Diabetes**								
Diagnosis	**0.0069**	**(0.0013, 0.0159)**	0.0005	(−0.0008, 0.0056)	0.0686	(−0.0069, 0.1373)	**0.0760**	**(0.0021, 0.1450)**
*Among diagnosed*								
Condition monitoring	**0.0120**	**(0.0002, 0.0343)**	−0.0025	(− 0.0183, 0.0033)	0.0910	(− 0.0271, 0.1804)	0.1005	(− 0.0194, 0.1956)
Health education	0.0042	(−0.0012, 0.0186)	−0.0042	(− 0.0196, 0.0011)	0.0056	(− 0.1004, 0.1172)	0.0056	(− 0.0954, 0.1155)
On medication	−0.0015	(− 0.0166, 0.0030)	0.0009	(− 0.0030, 0.0099)	0.0529	(− 0.0405, 0.1414)	0.0523	(− 0.0413, 0.1358)
Control	0.0019	(−0.0058, 0.0153)	0.0022	(−0.0020, 0.0218)	0.0008	(−0.1180, 0.0953)	0.0049	(−0.1130, 0.1037)
**Dyslipidemia**								
Diagnosis	**0.0055**	**(0.0003, 0.0099)**	0.0007	(−0.0003, 0.0023)	**0.1513**	**(0.1103, 0.2009)**	**0.1575**	**(0.1171, 0.2057)**
*Among diagnosed*								
On medication	−0.0045	(− 0.0137, 0.0025)	0.0002	(− 0.0012, 0.0057)	−0.0492	(− 0.1485, 0.0252)	−0.0534	(− 0.1318, 0.0283)
Control	−0.0020	(− 0.0144, 0.0064)	−0.0001	(− 0.0064, 0.0023)	−0.0235	(− 0.1111, 0.0752)	−0.0256	(− 0.1102, 0.0740)

^a^ Adjusting for age, sex, marital status, smoke, drink, social health insurance. Bolded coefficients are statistically significant at the P level of 0.05

As illustrated by Table 8, health checks could mediate the association between social participation and the diagnosis of hypertension, diabetes and dyslipidemia. The proportions of mediation were 17.5, 23.9 and 8.9%, respectively (see Table 8).

**Table 8:** Health checks as a mediating factor on the association between social participation and the diagnosis of hypertension, diabetes, and dyslipidemia, among Chinese adults aged 45 years and above^a^

Chronic conditions	Indirect effect via health checks		Direct effect		Total effect of social participation	
	Coefficient	95% CI	Coefficient	95% CI	Coefficient	95% CI
**Hypertension**	**0.0062**	**(0.0031, 0.0109)**	**0.0293**	**(0.0014, 0.0651)**	**0.0355**	**(0.0066, 0.0712)**
**Diabetes**	**0.0156**	**(0.0072, 0.0282)**	0.0499	(−0.0057, 0.1002)	**0.0654**	**(0.0090, 0.1201)**
**Dyslipidemia**	**0.0050**	**(0.0018, 0.0090)**	**0.0514**	**(0.0150, 0.0910)**	**0.0564**	**(0.0173, 0.0938)**

^a^ Adjusting for age, sex, marital status, smoke, drink, social health insurance. Bolded coefficients are statistically significant at the P level of 0.05

## Discussion

Our study investigated whether socio-economic inequalities exist in chronic disease management among Chinese adults, and whether the relationship between SES and chronic disease management is mediated by social capital. The hypotheses were supported by our data. Compared to urban adults, there were lower levels of health checks, diagnoses, and medical control among rural adults. Our study also provided evidence that social capital could mediate the association between SES and chronic disease management to a certain extent. This study suggests deeply reforming our social security system and enhancing the social capital construction to improve those low SES people’s health.

First, performance gap in the management of hypertension, diabetes and dyslipidemia was identified on the diagnose link. Although screening and routine management of hypertension and diabetes were freely provided since the National Public Health Service Program launched in 2009, the number of patients diagnosed remained far fewer than actual number of patients. In other words, the rates of cases identification among Chinese adults aged 45 and older with chronic conditions were unsatisfying. Among those with diagnosed chronic conditions, majority received routine monitoring and treatment, which revealed that the management of chronic conditions in China was rather a detection problem. The inadequate diagnosed pattern of the three chronic conditions and the insufficiency of essential health care were similar to previous studies [4, 33]. Specifically, the least common diagnosed diseases were dyslipidemia, followed by diabetes and hypertension. While the higher diagnosis rate might be accompanied with relative more management of chronic diseases as showed in Table 3. Therefore, it is imperative to elevate the diagnosis rate of chronic disease, particularly for dyslipidemia.

Furthermore, urban setting, higher education and higher economic status accounted for a higher rate of diagnosis. As a whole, the analysis revealed that only 37.3% of Chinese adults aged 45 and older had annual health checks, which may be a reason for the low diagnosis level. Further analysis showed subjects living in urban setting, with higher education attainment and economic status were more likely to have annual health checks, which may be beneficial to the higher diagnose rate.

Secondly, social capital could mediate the association between SES and chronic care along the continuum to a certain extent, especially for the aspects of diagnosis, health checks and health education. The mediation effect of social capital has also been found in previous study, which supported that social capital worked as a mediating link behind the socioeconomic difference in cardiovascular diseases [34]. Meanwhile, the mediating effects, which were all weak, were generally attributed to the structural social capital variable-social participation, but not cognitive social capital variable-perceived helpfulness. A prior study also suggested the mediating effect of social capital was different in two types, but cognitive social capital had stronger mediating effects on the association from education to depression, compared to structural social capital [35], since cognitive social capital is derived from psychosocial processes, while structural social capital often involves behavioral processes [20]. This also could explain why only structural social capital had significant mediating role on the relationship between SES and chronic disease management. Moore et al. indicated that subjects with lower SES are more likely to have lower social capital [36], as well as poor awareness and control of hypertension [37]. In addition, Western and Asian researches had proved the positive link between structural social capital and self-rated health [31, 38]. Combined with the results in the analysis, it implies that structure social capital mattered for management of chronic conditions, such as health checks, health education and diagnosis, and therefore is affecting the health status of patients with diabetes, hypertension and dyslipidemia. As showed in Table 8, health checks could also mediate the association between social participation and diagnosis of three chronic conditions, implying that the effect of social participation on management of chronic care may be due to the health-seeking desire of patients with chronic conditions. This finding was in line with previous research, old adults with higher degree of social participation tend to determine better self-care [39]. And people with high structural social capital could obtain more access to local health services and more information about health, and they may be more receptive to advice and norms about healthy behavior like getting routine health checks [40]. Besides, it is well known that regular health check-ups could be effective in earlier detection of disease [41]. Of note, the proportion of mediating effect of health checks on diagnosis of diabetes was the highest, following by diagnosis of hypertension and dyslipidemia. It suggested that individuals participated more social activities may be more likely to detect diabetes early through routine health checks, whereas diagnosis of hypertension and dyslipidemia may be directly affected by social participation. This may be because both the prevalence of hypertension and dyslipidemia were usually higher than the prevalence of diabetes [42], which means individuals may be more possibly to contact patients with dyslipidemia or hypertension during social participation, and thus learning more about related symptoms. It might be beneficial to diagnosis of dyslipidemia or hypertension.

Moreover, our results indicated that the proportion of having structural social capital is relative less than the rate of owning cognitive social capital, and the urban-rural difference in social participation is also larger than in perceived helpfulness. Considering the mediating role of structural social capital, it is need to increase social participation, such as interacting with friends, going to a community club or community-related organization, providing help to others who did not live with you and who did not pay you for help, doing voluntary or charity work, attending an educational or training course, and joining in a sport, social, or other kind of club, especially for over 45-year-old rural adults who have less social participation than urban adults. It could be beneficial to reduce the socio-economic inequalities in chronic diseases management and protect physic health of people with low SES. More importantly, no matter whether social capital was taken into consideration or not, there was no doubt that disparities of chronic conditions management exist among subjects with different levels of SES. In order to achieve the Sustainable Development Goals by 2030 and implement ‘Health in All Policies’ which was initially proposed in the declaration of Alma-Ata in 1978, it is necessary to eliminate the disparities of chronic conditions management by promoting social participation [43]. However, since the effects of social participation presented in this manuscript were generally weak on the management of chronic conditions, it seems that constructing the chronic care delivery system would have greater potential to achieve better chronic conditions management and better health status of certain patients.

Several limitations in this study should be acknowledged. First, this study is cross-sectional and we could not draw causal relationships of the mediation effects of social capital. In future, more longitudinal studies should further explore this important question. Second, we use the combined database from the CHALRS that contained the first anthropometric or blood measurement of the participants. Such a design could not yield nationally represented estimates, however, it could tease out the potential biases. Third, we only included CHARLS samples with blood pressure measurement, which generate a relatively high rate (19.1%) of missing values. Although we incorporated CHALRS weights that adjusted for missing values, which yields similar estimates against the unweighted results, we still cannot completely ignore the possibility that exclusion bias may have distorted the results and thus the finding of this study should be generalized with caution. Fourth, our analysis seemed to be the first attempt to investigate how social capital affects the effective coverage of chronic condition. However, the data had 2036 and 2350 of participants who had no anthropometric and venous blood measurement respectively. The missing values may not affect findings on the role of social capital, because covariates from the subsamples that we used did not vary substantially from the entire CHARLS sample. More importantly, the probabilities of missing for blood pressure and venous blood measurement were not associated with participant’s social capital.

## Conclusion

The findings in this article suggest that disparities of chronic conditions management exist among subjects with different levels of SES. Structural social capital works as a mediating role in the relationship between SES and chronic disease management. It could draw policy makers attention to the potential effect of social capital construction on improving the health of low SES people. The inclusion of low SES people in groups or organizations should be emphasized in the intervention strategies. Furthermore, a complete chronic care delivery system that can support for people in low level of SES should be an important goal of future policies.

## Data Availability

The datasets used and/or analyzed during the current study are available from the corresponding author on reasonable request.

## Abbreviations

NCDs: Non-communicable diseases
CHARLS: China Health and Retirement Longitudinal Study
SES: Social economic status
WHO: World Health organizations
PSUs: Primary sampling units
SBP: Systolic blood pressure
DBP: Diastolic blood pressure
HDL-C: High-density lipoprotein cholesterol
LDL-C: Low-density lipoprotein cholesterol
STATA: Software for Statistics and Data Science
NCMS: New Rural Cooperative Medical Scheme
URBMI: Urban Resident Basic Medical Insurance
UEBMI: Urban Employee Basic Medical insurance
GIS: Government insurance scheme
